# *Alternaria alternata* as endophyte and pathogen

**DOI:** 10.1099/mic.0.001153

**Published:** 2022-03-29

**Authors:** Mara DeMers

**Affiliations:** ^1^​ Plant and Microbial Biology Department, College of Biological Sciences, University of Minnesota, St. Paul, MN, USA

**Keywords:** taxonomy, systematics, symbiosis, fungi, species delimitation

## Abstract

*Alternaria alternata* is a common species of fungus frequently isolated from plants as both an endophyte and a pathogen. Although the current definition of *A. alternata* rests on a foundation of morphological, genetic and genomic analyses, doubts persist regarding the scope of *A. alternata* within the genus due to the varied symbiotic interactions and wide host range observed in these fungi. These doubts may be due in large part to the history of unstable taxonomy in *Alternaria*, based on limited morphological characters for species delimitation and host specificity associated with toxins encoded by genes carried on conditionally dispensable chromosomes. This review explores the history of *Alternaria* taxonomy, focusing in particular on the use of nutritional mode and host associations in species delimitation, with the goal of evaluating *A. alternata* as it currently stands based on taxonomic best practice. Given the recombination detected among isolates of *A. alternata*, different symbiotic associations in this species should not be considered phylogenetically informative.

## Introduction

Fungi are widespread and exhibit broad variation in nutritional mode, living both as symbionts and as saprophytes on organic matter [1–3]. However, fungal ecology and evolution interface in varied ways; nutritional mode does not appear to be correlated with phylogeny for most fungi [4–6], while other lineages seem constrained to a single lifestyle [6, 7]. In this review, I examine the relationship between nutritional mode and systematics in *Alternaria alternata,* a filamentous Ascomycete occurring on plants as a pathogen and endophyte, and in soil as a saprophyte [8].

*A. alternata* is currently considered to be a cosmopolitan species with a wide host range [9–13]. Because *A. alternata* is pathogenic on many important crop plants [14] but also lives in asymptomatic symbiosis as an endophyte of many plants [13, 15–20], it is necessary for plant pathologists to know whether a given strain of *A. alternata* indicates a threat to food safety or simply the presence of an endophyte [21–23]. Given the prevalence of endophytic *A. alternata* [13, 24], it is also vital for conservationists to understand if changes to the abiotic environment might cause asymptomatic infections of *A. alternata* to shift into parasitism, leading to further stress to vulnerable plant populations [25–29]. These questions, however, are difficult to address due to a history of unstable taxonomy in the genus *Alternaria* [10, 12, 23, 30–38].

Many taxa described as ‘cosmopolitan’ are associated with long histories of unsatisfactory taxonomic revision because they lack readily recognizable characters, so their diversity is not captured in their taxonomy [39–46]. Microscopic organisms in particular are said to suffer from the ‘low morphology problem’ [40], where few morphological characters might be used to broadly delimit species that are then widely applied [43, 44, 46]. Typically, subsequent phylogenetic analyses based on DNA sequencing of these broadly delimited morphospecies have resolved more narrowly defined species with smaller geographical and host ranges, as well as physiological characters that had been previously overlooked that serve to diagnose the newly delimited species (see meta-analysis [41–47]). Unfortunately, for *Alternaria* we see the opposite case; phylogenetic analyses of sequence data have found fewer monophyletic groups than morphospecies, and agronomically relevant host-specific toxins (HSTs) do not segregate with individual lineages [10, 12, 34, 36, 37, 48, 49]. Without a stable basis for species delimitation in *Alternaria*, it is unclear if traits such as host interactions and nutritional modes are phylogenetically conserved, or if *Alternaria* spp. are truly cosmopolitan.

The purpose of this review is to explore how *A. alternata* has been historically delimited, and how its varied host interactions have influenced the concept of species in this lineage. I begin with a brief history of endophytes, how early research into fungal endophytes and *Alternaria* intersect, and the close phylogenetic association of endophytism and parasitism. Then, I trace the taxonomy and systematics of *A. alternata* from its original morphological description to its most recent re-description and phylogenetic characterization. In particular, I highlight evidence for genetic incongruence and recombination in this lineage, and how this may facilitate transfer of genetic elements encoding HSTs. Finally, I discuss current best practices in fungal species recognition, and attempt to apply them to *A. alternata* as it is currently defined.

## A brief history of endophytes

### History and use of the term endophyte

Today, the term endophyte is generally applied to organisms that live inside of plants without causing symptoms of infection [25, 50–52], but its original usage was very different. Heinrich Friedrich Link first coined the term ‘entophytae’ in 1809, meaning merely any organism living inside a plant. His definition was intended for pathogens, since it was assumed that healthy plants did not harbour internal symbionts [53]. When Galippe reported finding bacteria and fungi inside of healthy plants in 1887 and posited that they might have a beneficial role, the application of ‘endophyte’ expanded to include the potential for mutualists and commensals [54]. When rhizobia and mycorrhizae were discovered in the 1880s [55, 56], they were allowed under this definition of endophyte as well, although having their own unique labels meant that ‘endophyte’ was only sporadically applied to them. It was not until a century later in 1986 that George Carroll restricted the use of the term endophyte to micro-organisms that cause asymptomatic infections within plant tissues, excluding pathogens and mutualists such as mycorrhizae and rhizobia [50]. Aside from minor considerations over the possibility of latent pathogens residing in asymptomatic plants [25], or non-mycorrhizal endophytes providing mutualistic benefits to their hosts [57–60], Carroll’s definition of endophytes is widely accepted today.

Research on fungal endophytes as they are currently defined grew out of the independent investigations of livestock toxicosis, plant pathogens and leaf litter decomposition [61–65]. Other papers occasionally reported the presence of endophytes indirectly, especially surveys of internal moulds in visibly healthy seeds, but surface-sterilization was not widely applied in seed fungus research so the status of these moulds as endophytes is sometimes uncertain [66–68]. The first major effort to isolate endophytes began in response to ryegrass staggers affecting livestock in New Zealand [61]. Fungal endophytes had previously been observed on the grass *Lolium perenne* in the late 1800s [69–71], and a fungal endophyte was implicated in the case of ryegrass staggers [61], but toxicity was not definitively assigned to a species of *Epichloë* (classified as *Neotyphodium* at the time) on *L. perenne* until 1981 [72]. Other *Epichloë* spp. have been isolated from other grasses [73–80], and their particular lifestyle of systemic infection and vertical transmission eventually led to the division of endophyte research between vertically and horizontally transmitted endophytes. We are interested in horizontally transmitted endophytes for the purposes of this review.

**Fig. 1.**
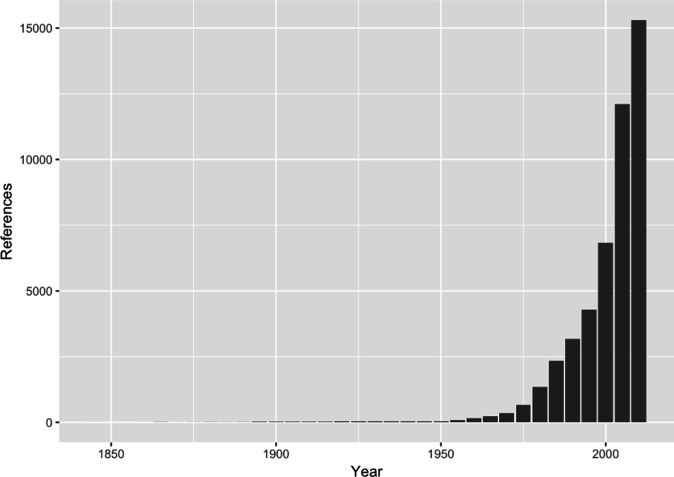
The number of papers referring to endophytes has rapidly increased, particularly due to the proliferation of environmental DNA sequencing beginning in the early 2000s. Yearly reference counts were retrieved by searching Google Scholar with the term ‘endophyt’ and binned in 5 year intervals.

Horizontally transmitted endophytes were originally sampled from healthy leaf tissue as a control to compare either diseased or decomposing leaves [62–65]. One research group focused on the endophytes of *Nicotiana* spp., particularly the difference between the endophytic *Alternaria* spp. abundant on these plants and the pathogenic *A. alternata* that caused leaf spot of tobacco on the same plants [16, 81–84]. Meanwhile, research on leaf litter decomposition [63, 65, 85] grew into an extensive exploration of the diversity and host affinities of endophytes of gymnosperms and other trees, due in large part to the work of George Carroll and his collaborators throughout the 1970s and 1980s [86–94]. These studies were the beginning of a rapid increase in the pace of endophyte research (Fig. 1), which was facilitated by use of DNA sequencing for fungal identification in the 1990s and environmental sequencing in the early 2000’s.

### Studies reporting *Alternaria* strains as endophytes

The earliest report of *Alternaria* isolated from asymptomatic plant tissue seems to be from an investigation of mycorrhizae published in 1905, where the endophytic *Alternaria* strain was isolated instead of the mycorrhizal target ([95] as summarized in [96]). *Alternaria* was also commonly found in studies targeting other fungi in the 1910s, including systemic endophytes of liverwort thallus [97], contaminants of wheat seeds [98], and mycorrhizae associated with orchid roots [96]. Surveys of seed fungi have frequently reported infestation by *Alternaria* spp., generally either *A. alternata* or its synonym ‘*A. tenuis*’ [17, 66, 68, 99–101]. Paul Neergaard, a major name in early *Alternaria* taxonomy, was better known as a seed pathologist [102].

Among studies purposefully sampling endophytes, a strain of *Alternaria* was first isolated as one of the fast-growing fungi that overgrew the elusive *Epichloë* and complicated the search for the causal agent of ryegrass staggers [61]. *Alternaria* spp., likely *A. alternata* as currently defined, were the most abundant endophytes isolated from *Nicotiana* spp. by Spurr and Welty [16]. After pathogenicity assays confirmed that endophytic isolates were unable to cause tobacco leaf spot, Spurr and Welty speculated that the abundance of endophytic *Alternaria* isolates on tobacco was contributing to inconsistencies in species delimitation for the pathogenic *A. alternata* isolated from tobacco [16], which have since been renamed *A. longipes* [10].

Aside from tobacco, endophytic *A. alternata* has been reported from a variety of crop plants, including strawberry, apple, rapeseed, soybean, rice and citrus [48, 103–109]. Notably, *Alternaria* spp. including *A. alternata* are also considered pathogenic on these plants [14]. Incidence of isolation varies among host species and among different studies on the same host [110, 111]; occasionally *Alternaria* strains are the most abundant endophytes isolated [16, 24, 112], but usually they are detected in 1–10 % of samples [15, 17–19].

### Phylogenetic analysis of endophytic lifestyle

Endophytism is common among Kingdom Fungi and has apparently evolved multiple times [4, 6]. In a phylogeny of the Ascomycota, frequent transitions between endophytism and parasitism were predicted by ancestral state reconstruction [4]. Transitions from parasitism to endophytism have been detected within individual genera, even multiple times [5, 7]. Transitions between endophytism and saprotrophy appear to be less common [4], and biotrophic pathogens do not appear to revert to other nutritional modes once they have evolved [6]. Ancestral state reconstructions of nutritional modes are complicated by uncertainty in assigning a species or isolate to a nutritional mode based on observations in nature, as well as species that encompass several different lifestyles [4].

It has also been proposed that endophytic fungi are pathogens or saprophytes waiting for ideal conditions to emerge [5, 113–115]. Indeed, some endophytes may also function as saprophytes since they have been isolated from both living and dead host tissue [116–120], and others are capable of causing disease on their endophytic host [121]. More generally, many fungi may cause endophytic or pathogenic infections depending on host identity, host condition and abiotic factors [26, 29, 115, 122–126]. Speciation based on endophytic specialization in asexual lineages arising from pathogens has occurred in vertically transmitted *Epichloë* on ryegrass [127], and may be occurring in an exclusively endophytic, asexual lineage of *Verticillium dahliae* on mustards [7], but it should not be considered the norm for horizontally transmitted endophytes.

### Taxonomy and systematics of the genus *Alternaria*

### Description of *Alternaria* amongst the ‘waste basket assemblage’

The first species in the genus *Alternaria*, *A. tenuis* was described from dead plant material by Nees von Esenbeck in 1816 [128], without sufficient characters for delimitation of phenotypically similar species or a surviving type specimen [13, 30, 31, 129]. The situation was complicated by Fries initially rejecting Nees' description while describing the highly similar genus *Macrosporium*, and then electing to synonymize *A. tenuis* under the name *Torula alternata* in 1819 [30, 130]. After Chevallier reported conflicting spore morphology between *Alternaria* and *Torula* [131], Fries finally accepted its generic status, but maintained that *Macrosporium* was a separate genus [31, 132, 133]. The distinction of these two genera was widely criticized due to overlap in morphological characters and host associations [30, 31, 129, 134, 135], and most species previously ascribed to *Macrosporium* are now categorized under *Alternaria* [9, 136].

Eventually the type species for *Alternaria* was formally established as *Alternaria alternata* (Fries) Keissler (1912), despite the apparent precedence of the name ‘*A. tenuis*’ for the same taxon as described by Nees [128]. This inconsistency is due to the status of *‘A. tenuis*’ as an ‘invalid pre-starting date epithet’ based on the International Code of Botanical Nomenclature, and therefore illegal [32, 129]. As a result of this taxonomic technicality, ‘*A. tenuis*’ remained the more popular epithet applied to *A. alternaria* specimens for decades [31, 137], including one particularly egregious use of ‘*A. tenuis* auct. sensu str. cf. Neergaard (1945)’ by Yu, Mathur and Neergaard in 1982 [138]. Use of *A. alternata* was popularized by Emory Simmons, who spent decades cataloguing *Alternaria* spp. based on extremely fine morphological examinations beginning in the 1960s [32, 139]. However, ‘*A. tenuis*’ still persists in modern publications, likely due to GenBank records that still carry the old name [140].

Simmons, in addition to being the foremost expert on *Alternaria* morphology, was also extremely disdainful of Fries and his contributions to *Alternaria* taxonomy [141]. Simmons claimed that Fries had a low opinion of microfungi and relied on ‘published observations which often were as poor as or worse than his own,’ and that his classifications were no better than ‘historical fiction.’ [141]. In defence of Fries, fungi that lack a known sexual stage (‘anamorphs’ vs. sexual ‘teliomorphs’) are character-poor and have historically proven difficult to categorize [142]. Later authors declared that anamorphs such as *Alternaria* spp. were at best a confusing problem and at worst not worth characterizing, and lumped them all into the unnatural category of ‘Fungi Imperfecti’ [141–143]. Saccardo’s classification system for the Fungi Imperfecti, based on spore morphology and specific host interactions, was a practical solution for classifying plant pathogens that broke down almost immediately when every soil fungus without a known sexual stage was thrown into the ‘waste basket assemblage’ of Fungi Imperfecti [142]. By the time over 30 thousand ‘imperfect’ fungal species had been unceremoniously dumped into the bin, a great deal of inertia stood in the way of their ever being systematically reclassified [144].

## Generic delimitation and species concepts in *Alternaria*: a problem with unsatisfying solutions

Unfortunately for the taxonomists of the 1900s, any alternative to Saccardo’s strategy for classifying microfungi without a known sexual phase was also inevitably morphological [142], and morphological classifications can be subjective [30]. Several camps of taxonomists (Elliott, Young, Wiltshire, Neergaard, Joly, Simmons, later Simmons, Roberts, Nishimura and colleagues) spent the bulk of the 1900s trying to circumscribe the genus *Alternaria* and catalogue its species using whatever physical and biochemical characters they could reliably detect. These camps rarely agreed.

The early period of generic circumscription featured arguments concerning synonymizing highly similar genera and the reliability of spore measurements and host ranges as informative characters [30, 145]. Elliott explicitly studied spore measurements by fixing several specimens and sending them to colleagues, and determined that variability between microscopists was greater than published intra-species variability [30]. Without an acceptable alternative forthcoming, species description based on spore measurements persisted [135, 146]. Species were frequently described, synonymized, and shuffled among genera including *Alternaria*, *Macrosporium*, *Stemphylium* and *Pleospora* (there was also, briefly, an expectation that all *Alternaria* spp. would eventually be revealed as anamorphs of *Pleospora*) [30, 32, 129, 134, 135, 137, 145, 147]. Disagreements over taxonomic classifications appear to have become somewhat heated (see in particular Joly’s take on Neergaard [147] and Simmons’s review of Joly [148]), although they may have been more civil in person than they were in print. The status of these genera could not satisfactorily be settled until the application of molecular methods [9, 21, 136]; in the meantime, the focus of taxonomists shifted to species delimitation.

*Alternaria* species can be broadly split into two categories: large spored species, which are relatively simple to diagnose due to their distinctive morphologies and stable host ranges, and small spored species, which are not [36, 149]. Among the small spored species, the group currently called *A. infectoria* was often treated separately in *Alternaria* taxonomic studies due to its sexual stage (otherwise not observed in *Alternaria*) identified as a species of *Pleospora* [21, 30]. The remainder of small spored *Alternaria* are currently classified as Section Alternaria [9–12, 136] and include *A. alternata*, *A. arborescens* and a wide variety of presumed host-specific plant pathogens that have been described as both species and as pathotypes or formae speciales of *A. alternata* [10, 34, 150–152]. Section Alternaria, being the figurative squeaky wheel, has received the bulk of the taxonomic revision.

Initially, it was difficult to positively identify or describe new species similar to *A. alternata*, the type species of *Alternaria*, because no type material was available for comparison [129]. This changed with the discovery of collections originally identified as *A. tenuis* by Nees that had been stored in Persoon’s herbarium, allowing Simmons to designate a formal epitype for *A. alternata* in 1967 [32]. Emboldened by the certainty of a good type specimen, Simmons proceeded to describe *Alternaria* spp. for the next 40 years [153–160], recognizing a total of 275 species with the publication of a monograph in 2007 [138]. These species descriptions were based on morphological observations (patterns of conidiophore development under tightly regulated culture conditions) and pathogenicity studies, going well beyond simple spore measurements [35], although some of the characters (particularly conidiophore development) were considered difficult to apply by non-experts [149]. Simmons' concepts of species were generally considered to be very narrowly defined [9, 36].

Opponents to Simmons' narrow species descriptions questioned the biological relevance and diagnostic value of the morphological characters he favoured [34]. Slifkin used electron microscopy to measure the variance of spore and conidiophore morphology within isolates and concluded that either these characters were not diagnostic, or that most of the species she examined should be synonymized [161]. Meanwhile, Nishimura and colleagues had begun describing pathogenic isolates as pathotypes of *A. alternata*, as these isolates were morphologically indistinguishable from non-pathogenic *A. alternata* aside from the production of key host-specific toxins (HSTs) that could be lost in long-term culture [33, 161–163]. Simmons, conversely, considered production of HSTs to be a basis for species differentiation [160]. More recent studies making use of DNA sequencing have supported a more broadly delimited *A. alternata* with pathotypes distinguished by HSTs, although the systematics of Section Alternaria is still the subject of study [10, 12].

### *Alternaria* in the sequencing era (1995 to present)

**Fig. 2.**
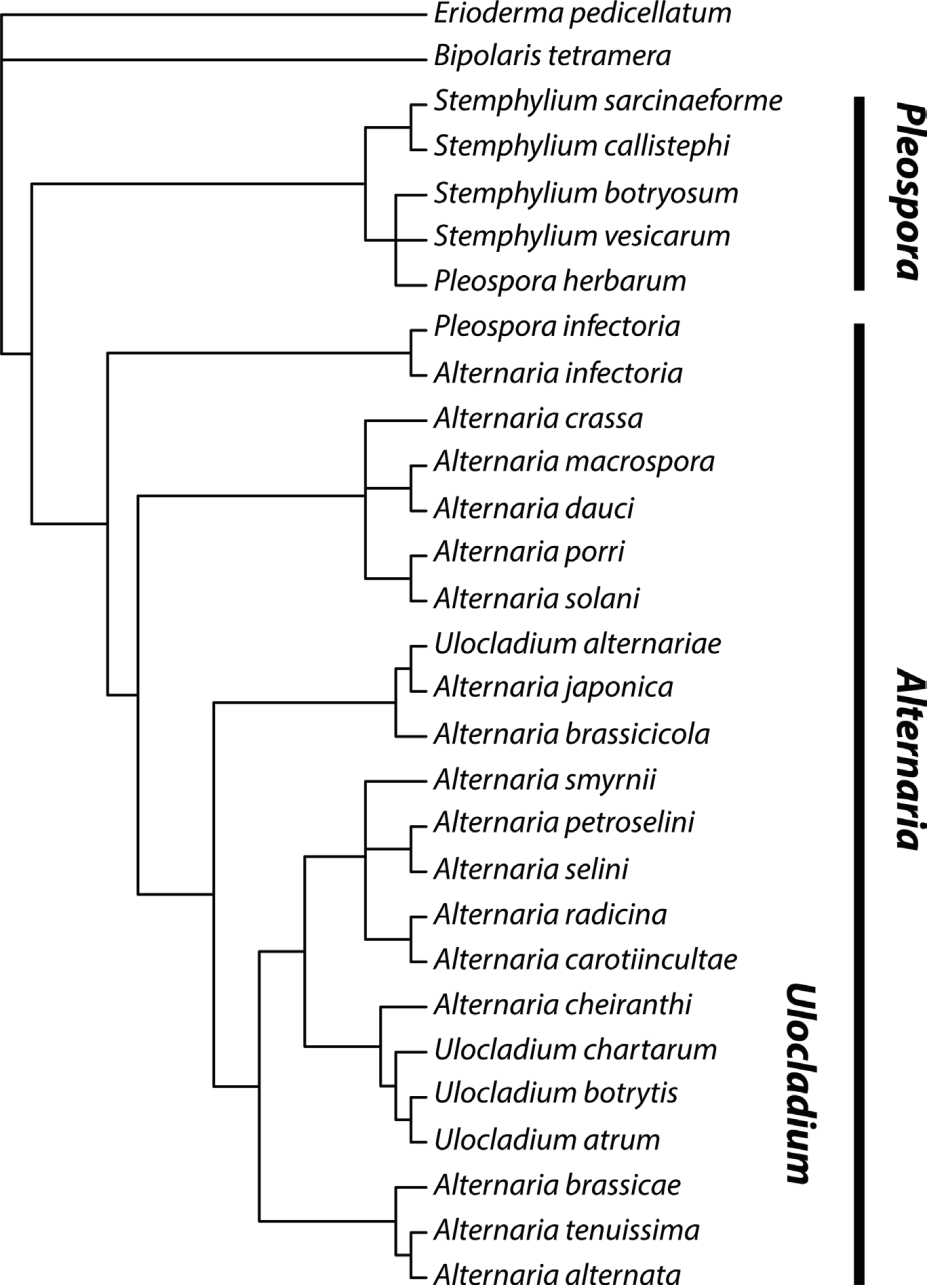
Relationships between species of *Alternaria*, *Ulocladium*, *Stemphylium* and *Pleospora* as determined by Pryor and Gilbertson [21]. *Ulocladium* spp. are nested within the *Alternaria* lineage. *Pleospora* (here containing several *Stemphylium* spp.) appears as sister to *Alternaria*, except for a single individual assigned to ‘*P. infectoria*’ in the *A. infectoria* clade. Redrawn based on phylogeny constructed using neighbour-joining and parsimony analysis of nuclear ITS/5.8S rDNA sequences [21].

**Fig. 3.**
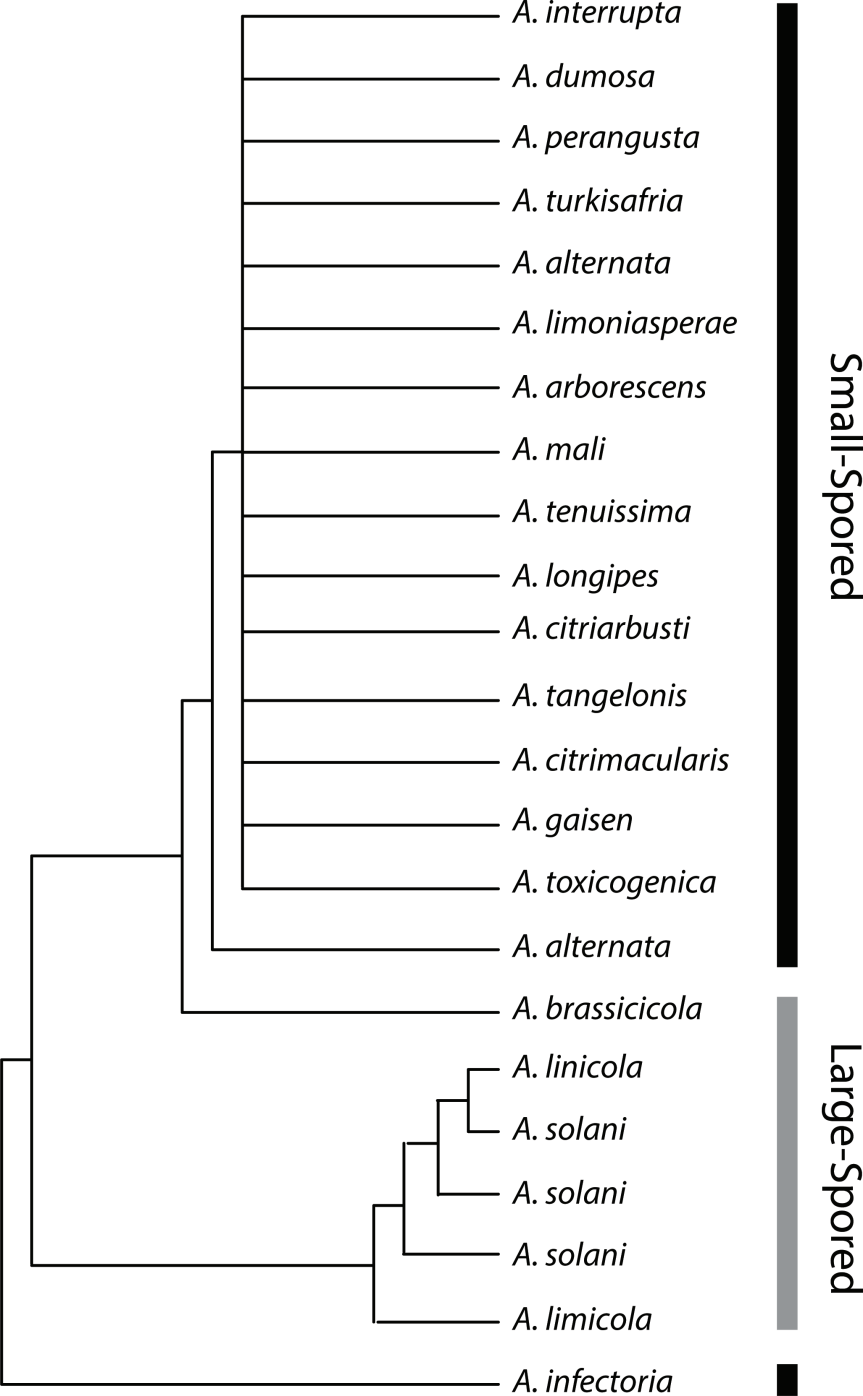
Spore size trait (large or small) mapped onto *Alternaria* spp. based on phylogeny constructed by Peever *et al*. [36] in an analysis of citrus pathogens. All small-spored isolates from citrus were indistinguishable from *A. alternata* using mitochondrial RNA large subunit (mtLSU) sequence data. Redrawn based on phylogeny constructed using maximum likelihood analysis of mtLSU sequences [36].

DNA sequencing to characterize isolates of *Alternaria* appears to have begun in earnest in 1995. Jasalavich *et al*. [164] reported the first molecular evidence that *Alternaria* belongs in the Pleosporaceae, and found that *Alternaria* was closely related to *Stemphylium* and *Pleospora*, in support of morphological evidence. In the same year, Kusaba and Tsuge [34] constructed a phylogeny of HST-producing *Alternaria* from ITS sequences and could not distinguish HST-producers from non-pathogenic *A. alternata*, in agreement with the recommendations of Nishimura *et al*. [33] and previous findings based on restriction fragment length polymorphisms (RFLPs) [165, 166]. Additional sequence-based phylogenetic studies found similar results, clustering several genera (*Macrosporium*, *Stemphylium*, *Ulocladium*, *Lewia*, *Nimbya*) closely with *Alternaria* and failing to support species status for many morphospecies (Fig. 2), particularly pathogens of citrus (Fig. 3) [21, 27, 36, 37, 149, 167].

**Fig. 4.**
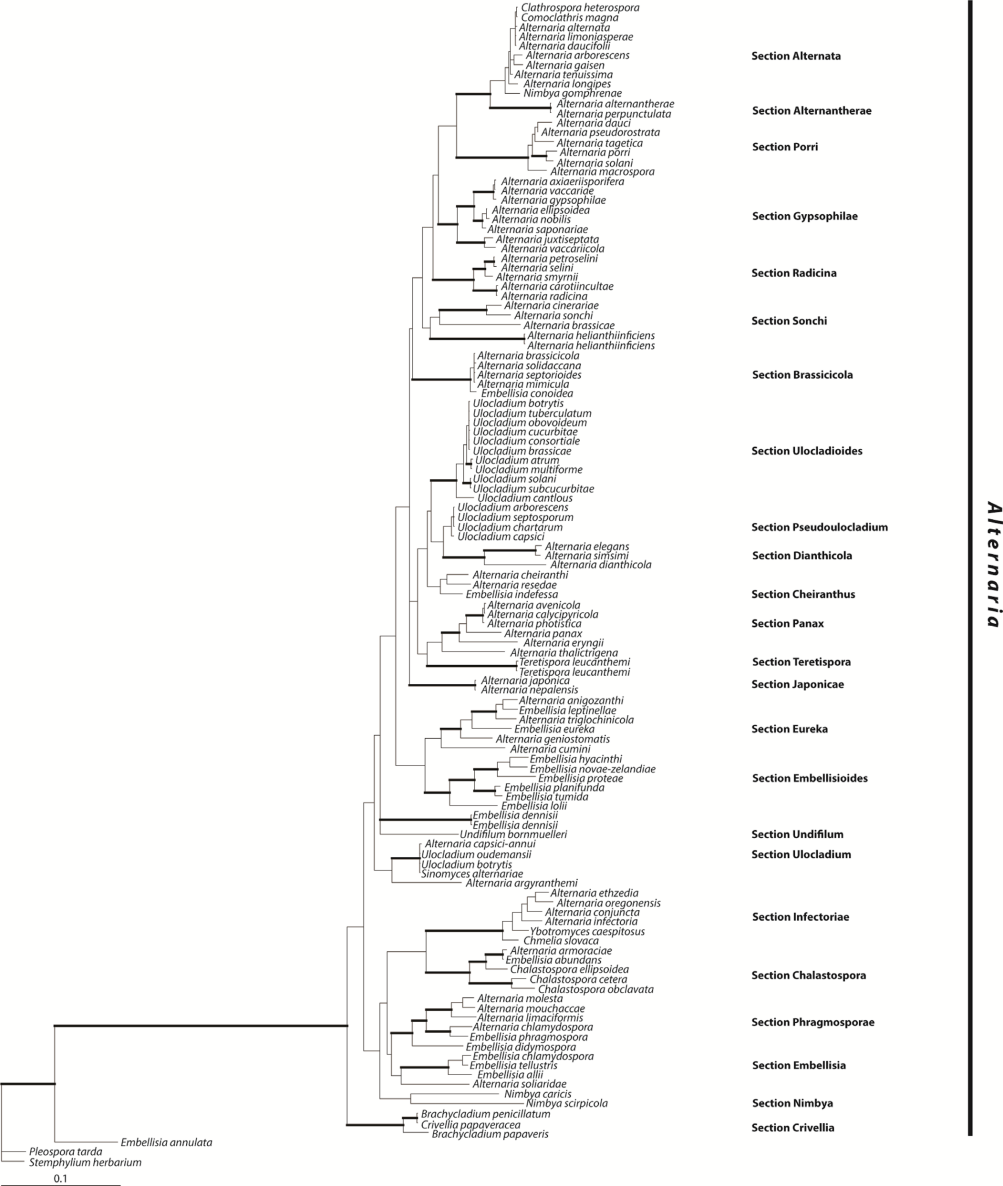
Bayesian 50 % majority rule consensus tree based on three loci showing the phylogeny of the genus *Alternaria* as defined by Woudenberg *et al*. [9]. Sections proposed by Woudenberg *et al*. are shown at the right. Adapted from Fig. 1 of [9] with permission from the authors.

For several years, authors seemed hesitant to address the increasing number of genera closely aligned to *Alternaria*, unless it was to propose creating more. *Lewia* was established to house *Pleospora* spp. with *Alternaria* anamorphs [21], and *Undifilum* was established for swainsonine-producing mutualists of locoweeds [168]. Maintaining the status of these genera alongside a monophyletic *Alternaria* meant excluding all *Alternaria* spp. with a sexual stage; this would have required reclassifying the medically important and widely applied epithet *A. infectoria* (Fig. 4, Sect. Infectoriae) [149]. Lawrence *et al*. [169] proposed reclassifying *Nimbya* and *Embellisia* as *Alternaria*, since their status as separate genera was only supported by morphological characters, but later chose to discuss *Alternaria* as polyphyletic rather than come down on either side of including or excluding *A. infectoria* [136]. Finally, Woudenberg *et al*. redefined *Alternaria* to definitively include *A. infectoria*, as well as all allied genera that would otherwise be rendered paraphyletic (Fig. 4) [9].

### Recombination among lineages of *Alternaria alternata*

**Fig. 5.**
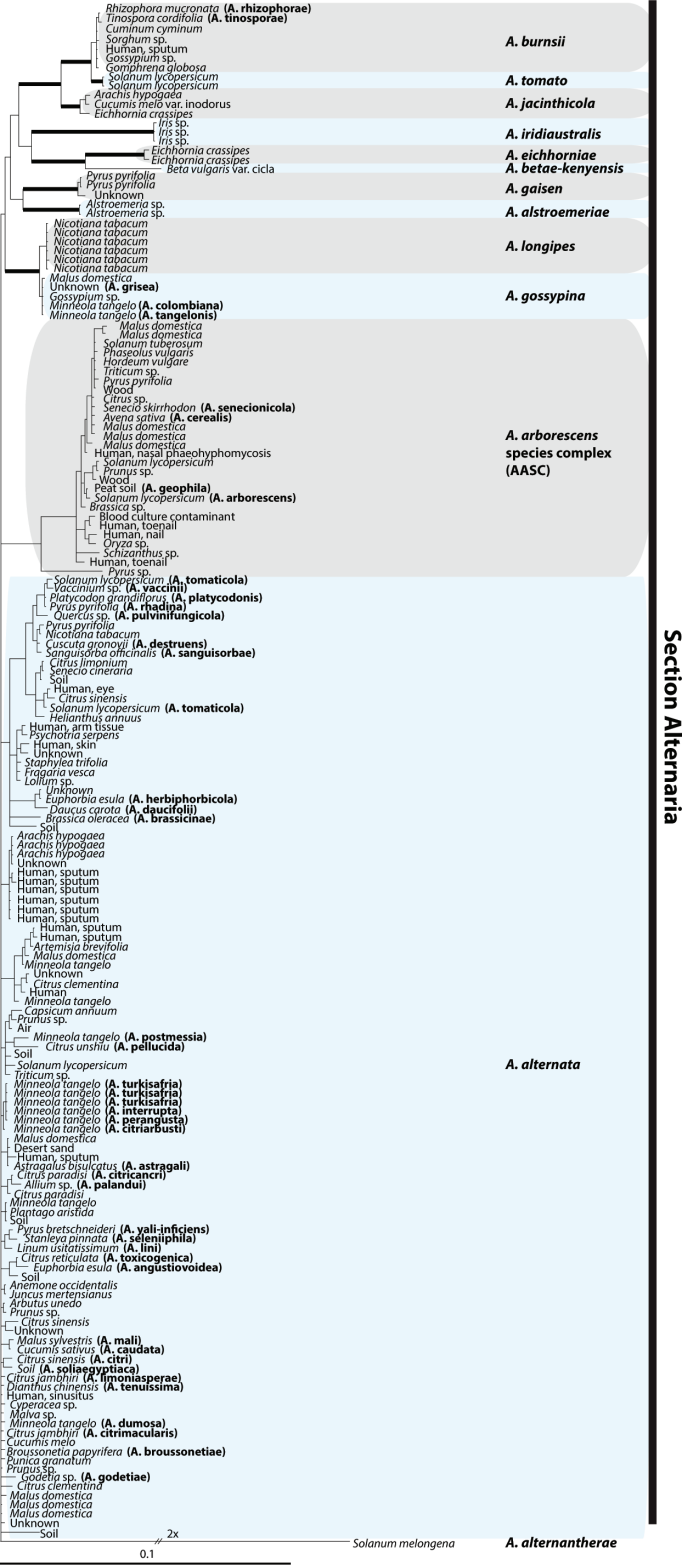
Bayesian 50 % majority rule consensus tree based on seven loci, showing isolation sources of *Alternaria* spp. in Section Alternaria as re-defined by Woudenberg *et al*. [10]. Accessions with names synonymized by Woudenberg *et al*. are noted in parentheses. Retained species are delimited by highlight (grey or blue) with the epithet labelled at the right. Adapted from Fig. 2 of [10] with permission from the authors.

The redefinition of *Alternaria* by Woudenberg *et al*. resolved deeper systematic issues, but could not address *A. alternata* and its purported pathotypes beyond placing them into a single section, Sect. Alternaria [9]. Further efforts to characterize Sect. Alternaria have generally supported the use of pathotypes to describe isolates that produce HSTs rather than elevating them to species, and have synonymized more species under the name *A. alternata* (Fig. 5) [10, 11, 48]. Different studies have reported some support for an *A. arborescens* species complex separate from *A. alternata* based on morphological and molecular characters [10, 11, 14], but the entire section seems to be characterized by a common metabolic profile [14, 22, 23, 170]. Based on these shared metabolites, Patriarca *et al*. [23] suggested that Section Alternaria should be considered a threat to food safety regardless of species delimitation within the section.

Several studies of taxa synonymized under *A. alternata* by Woudenberg *et al*. [10] have found evidence for gene tree discordance and recombination, suggesting that sexual or parasexual (nuclear fusion followed by haploidization) reproduction occurs in this lineage [38, 48, 49]. The presence of both mating type idiomorphs (highly diverged alleles) in roughly equal frequencies is considered good evidence for the maintenance of sexual reproduction in Ascomycetes [171], and both mating types have been sampled from *A. alternata* populations [12, 48]. Parasexual reproduction has been implicated as the mechanism of recombination in *A. solani* [172], and either method of genetic exchange could explain the signals of recombination observed in *A. alternata* [48].

Given the history of exchange across lineages, HSTs are not phylogenetically informative, but they do inform potential pathology. Horizontal exchange is facilitated by the location of HSTs on conditionally dispensable chromosomes (CDCs), which may segregate separately from the rest of the genome and can be lost or gained over time [173–180]. Recombination through sexual or parasexual reproduction may facilitate transfer of CDCs among lineages [12, 48, 181]. While screening for CDCs that carry HSTs seems to be the best course of action for the purposes of detecting economically important pathogenic isolates [12, 182], CDCs might be found in unexpected places. Strains capable of producing HSTs have been isolated as endophytes from non-target hosts [151, 183, 184], suggesting that specificity resides in the host-toxin interactions rather than as a characteristic of the toxin itself [150].

### The concept of species in Fungi and current best practices

An essential goal of taxonomy and systematics is to reliably place individual organisms into categories such that we can make predictions about their structure and physiology. Taken to either extreme, too strict or too broad, and these categories become impractical: extremely strict categories will be highly predictive but rarely applicable, while broad categories can be widely applied but allow few meaningful predictions of biological form or function. Species, then, are constructs representing the smallest category of organisms allowing us to make biologically relevant predictions [185, 186]. The practical application of taxonomy is deciding how dissimilar two species need to be.

Underlying all modern concepts of species is the Evolutionary Species Concept [185, 187], the idea that species should be monophyletic groups and share both evolutionary history and unique, diagnosable characters [185–188]. Less theoretical species concepts help us to recognize species under this guiding principle, and establish boundaries between species. The most widely used methods of species delimitation in fungi have historically been the Morphological Species concept [189] and the Biological Species Concept [190, 191], particularly due to the requirement for morphological descriptions when describing new species of fungi [46]. Species defined solely by morphology tend to be too broad and can be confounded by convergent evolution [42, 44–46], whereas biological species cannot be defined in fungi without a sexual stage [186], but both of these concepts are valuable for species recognition if species are first defined by another method [46, 47]. Early definitions in the genus *Alternaria* were based entirely on morphology [30–32, 128–134].

Current best practice in fungal species delimitation is use of phylogenetic species based on Genealogical Concordance Phylogenetic Species Recognition (GCPSR) [171, 192]. By this method, species boundaries are defined at the point of transition between gene tree concordance and conflict [187, 193–196]. This method is broadly applicable despite the prevalence of asexual reproduction strategies in fungi because at least low levels of recombination occur even in largely clonal lineages, and purely asexual lineages are extremely rare (reviewed in [171]). Recent systematic treatments of the genus *Alternaria* and Section Alternaria have relied on GCPSR [9–11].

## Conclusions

Woudenberg *et al*. [10] last defined *A. alternata* in 2015 based on whole-genome sequences as well as a multi-locus phylogeny, synonymizing most of its previous pathotypes and morphologically similar taxa (Fig. 5). CDCs carrying HSTs segregate independently among lineages within this species, likely through horizontal transfer or recombination via sexual or parasexual reproduction [173–180], and should not be considered diagnostic of monophyletic groups. More recently, authors have referred to this group as a species complex (or the ‘tenuissima clade’ [12]), but based on best practice in species delimitation, the re-definition by Woudenberg *et al*. [10] presents an adequate representation of the taxonomy of this lineage. *A. alternata* is, in fact, a cosmopolitan species with a wide host range encompassing multiple nutritional modes.

Given the phylogenetic basis of endophytism and varied lifestyles of many fungal species [26, 29, 115, 122–126], as well as several instances of pathogenicity in multiple lineages of *A. alternata* not associated with HSTs [27, 36, 37, 48, 49, 197], I suspect that all lineages of *A. alternata* are capable of both endophytism and at least mild pathogenicity. The broad suite of metabolites characteristic of Section Alternaria would likely support a capacity for varied host interactions and nutritional modes [14, 22, 23, 170]. In short, there is no evidence that particular lineages of *A. alternata* are genetically constrained to endophytism or parasitism on particular plants, aside from specificity imparted by production of HSTs.
